# Surface Defects Detection of Stamping and Grinding Flat Parts Based on Machine Vision

**DOI:** 10.3390/s20164531

**Published:** 2020-08-13

**Authors:** Hongzhi Tian, Dongxing Wang, Jiangang Lin, Qilin Chen, Zhaocai Liu

**Affiliations:** School of Electromechanical and Automotive Engineering, Yantai University, Yantai 264005, China; tianhz1994@163.com (H.T.); forstudy_lin@163.com (J.L.); kylin123_chen@163.com (Q.C.); zhaocai_liu@163.com (Z.L.)

**Keywords:** machine vision, image processing, feature extraction, multi-angle illuminations, detection

## Abstract

Currently, surface defect detection of stamping grinding flat parts is mainly undertaken through observation by the naked eye. In order to improve the automatic degree of surface defects detection in stamping grinding flat parts, a real-time detection system based on machine vision is designed. Under plane illumination mode, the whole region of the parts is clear and the outline is obvious, but the tiny defects are difficult to find; Under multi-angle illumination mode, the tiny defects of the parts can be highlighted. In view of the above situation, a lighting method combining plane illumination mode with multi-angle illumination mode is designed, and five kinds of defects are automatically detected by different detection methods. Firstly, the parts are located and segmented according to the plane light source image, and the defects are detected according to the gray anomaly. Secondly, according to the surface of the parts reflective characteristics, the influence of the reflection on the image is minimized by adjusting the exposure time of the camera, and the position and direction of the edge line of the gray anomaly region of the multi-angle light source image are used to determine whether the anomaly region is a defect. The experimental results demonstrate that the system has a high detection success rate, which can meet the real-time detection rEquation uirements of a factory.

## 1. Introduction

With the mass production of parts, the inspection of product quality is very important during the process of parts production. The traditional detection methods of surface defects rely on manual detection, and they suffer from an inherently low degree of automation and low detection efficiency, and the entire inspection process is subjective. With the development of automation technology, the detection of surface defects of parts has gradually changed from manual detection to machine detection, in which machine vision is a very popular detection method [1,2,3].

Machine vision detection technology has become a newly developed automatic intelligent detection method in recent years. It is a new detection technology which relies on computer science and uses industrial Equation uipment instead of human eye to judge product quality. With its objective, efficient, accurate, non-contact, low-cost, easy to maintain and other characteristics, it has become a more ideal technical means of monitoring [4,5]. As a promising and non-destructive measurement technique, machine vision is more widely used in surface defects detection of parts, and many experts and scholars have also studied and explored the application field [6,7,8,9]. Zhou [10] extracted the candidate defect regions from the vehicle body image by a multi-scale Hessian matrix fusion method, and candidate defect regions are classified into pseudo-defects, dents and scratches by feature extraction (shape, size, statistics and divergence features) and a support vector machine algorithm. Huang [11] developed a vision sensor based on the principle of laser triangulation. Through the visual analysis of the acquired 3D profiles of the weld, the presences as well as the positions and sizes of the weld defects can be accurately identified and therefore, the non-destructive weld quality inspection can be achieved. Roberto [12] using a novel strategy for feature extraction from Gabor filters combined with three different classification techniques to preprocess, segment, and classify the acquired images. Xu [13] proposed a method based on polarized light-filtering and optimized the structure parameters of the lighting system. The method was more effective compared with conventional piston surface defects defection methods. Wu [14] proposed a hybrid method for the automated visual inspection of the solder bumps. Then in the training stage, several template integrated circuit boards (ICBs) were used to find the positions and characteristics of solder bumps. Finally in the inspection stage, the defects could be detected. Min [15] designed an image acquisition device Equation uipped with LED auxiliary light source and shading box and proposed a method based on machine vision for real-time detection of track surface defects and a basis system of machine vision detection. Chen [16] designed a lighting system with dual light sources located at both sides of the inspected balls. The images are enhanced by using the closing top-hat operation and variance transform. Using binary large object analysis can achieve real-time detection. Emam [17] investigated straightness of sides, rectangularity, center curvature, side curvature, and warpage parameters, an experimental setup based on the stereo vision was established for extracting and analyzing the tile dimensions. Urbonas [18] adopted the faster R-CNN method to evaluate the quality of wood veneer and addressed the problem as a small data problem and apply data augmentation and transfer learning to improve the classification results. Zhang [19] designed a system by multiple vision machines, which guarantees high-speed defect detection on multiple surfaces of three-dimensional magnetic rings and two image processing algorithms are proposed, namely, the image edge removal algorithm (IERA) and magnetic ring location algorithm (MRLA), respectively, which effectively solve the defect segmentation problem. Jeyaraj [20] considered a characteristic of texture, namely its colour. A deep convolutional neural network is formed to learn from the training phase of various defect data sets. In the testing phase, the authors have utilised a learning feature for defect classification. Marchewka [21] presented a state-of-the-art framework for structural health monitoring of steel bridges that involves literature review on steel bridges health monitoring, drone route planning, image acquisition, identification of visual markers that may indicate a poor condition of the structure and determining the scope of applicability.

In order to realize the automatic detection of surface defects of stamping grinding flat parts, this paper aims to design an automatic detection system of stamping grinding flat parts based on machine vision. In addition, in order to meet the real-time detection rEquation uirements of the factory, the innovative solution will be put forward from two aspects of illumination mode and an image-processing algorithm, and the experimental platform will be built for experimental verification. The process is as follows: firstly, the parts are located and segmented according to the image of plane light source, the region of interest is divided into two parts: center panel region and edge annular region, and the defects are detected according to the gray anomaly; secondly, according to the surface of the parts reflective characteristics, the influence of the reflection on the image is minimized by adjusting the exposure time of the camera, and the position and direction of the edge line of the gray anomaly region of the multi-angle light source image are used to determine whether the anomaly region is a defect. Finally, the experimental results contrast with the manual detection results and demonstrate that the detection accuracy of the system can reach 100% and the identification accuracy of the system can reach 98.6%. [22,23,24] The comparison to other works is as shown in Table 1.

## 2. Detection System Framework

**Figure 1:**
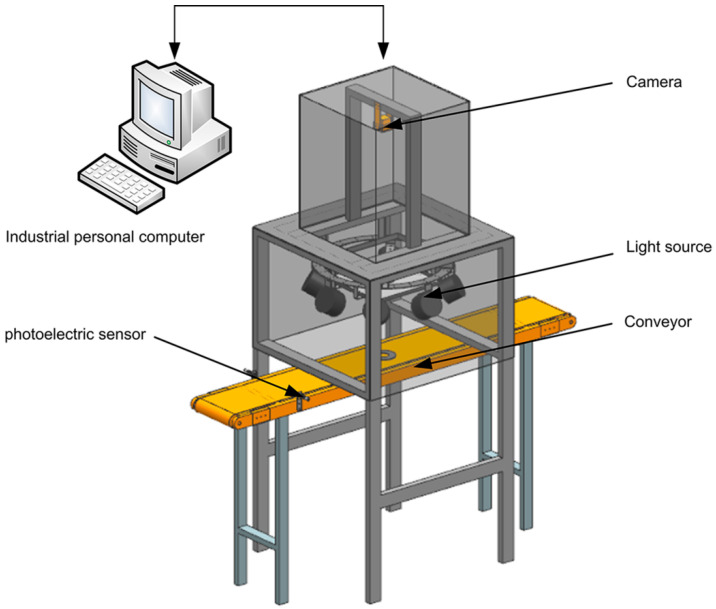
Illustration of hardware in the system.

This system mainly includes three modules: parts conveying module, image acquisition module and defects detection module. (1) The parts-conveying module is used to realize the movement control and sorting of parts. (2) The image acquisition module is composed of a camera, light source and photoelectric sensor, which is used to obtain high-quality images in the process of moving parts. (3) The defects detection module is used to process the collected high-quality images and realize the detection and recognition of the surface defects of parts. The specific process is as follows: the parts is transmitted by the conveyor belt, when it passes through the photoelectric sensor, the signal is transmitted to the console, and the image acquisition module is controlled by the console to collect the parts image. Finally, the defect detection module detects and identifies the defect and displays the result in the human–machine interface. The detection system framework as shown in Figure 1.

## 3. Imaging System

**Figure 2:**
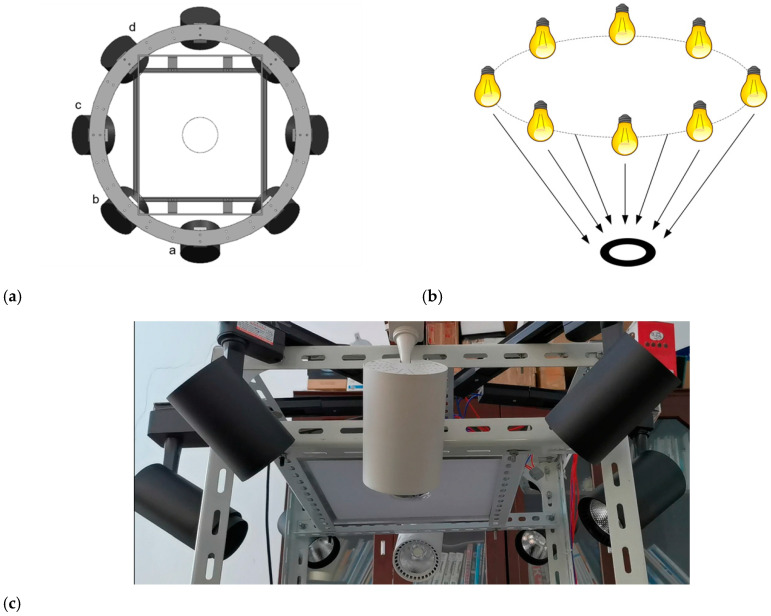
Imaging system. (**a**) The model of light source; (**b**) illumination schematic diagram; (**c**) light source device.

The commonly used plane light source has no directionality, the illumination is even, the collected image is not affected by the direction of the grinding marks, the whole region of the parts is clear and the outline is obvious, but it is difficult to highlight the tiny defects, and the image processing is prone to missed detection. In order to realize the detection and recognition of various types of defect on the surface of stamping parts, two lighting modes are designed in this system, which are plane light source with uniform illumination and four groups of multi-angle light sources. A 300×300 (mm) light-emitting diode (LED) flat light is selected as the plane light source. The LED flat light is parallel to the upper surface of the conveyor belt, 40cm away from the conveyor belt. The camera is located in the center of the flat light, and passing through the flat lights, and the camera spindle is vertical to the flat light. Eight LED spotlights are arranged in a ring around the camera to form multi-angle light source. The angle between the two adjacent LED spotlights is 45 degrees, and the main light of each LED spotlight is 45 degrees to the surface of the conveyor belt. The two LED spotlights with opposite directions are a group of angle light sources. The grouping is shown in Figure 2a. The camera and light source are covered in a closed black shell to avoid the interference of ambient light, and the black conveyor belt is selected as the shooting background to separate the parts area and background area during image processing. The image system is shown in Figure 2.

## 4. Defects and Challenges

In the process of stamping parts, many kinds of damages are usually caused to the parts, among which the more common ones are stain, misrun, indentation, edging and scratch.

**Figure 3:**
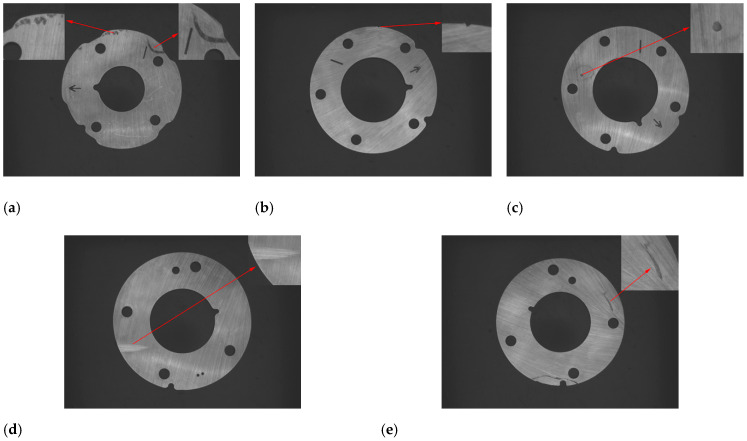
Five kinds of common defects. (**a**) stain; (**b**) misrun; (**c**) indentation; (**d**) edging; (**e**) scratch.

1. The stain is caused by impurities mixed in the raw material and randomly distributed on the surface of the parts, as shown in Figure 3a.
2. The misrun is caused by the collision of parts, and it is mostly distributed on the edge of the parts, as shown in Figure 3b.
3. The indentation is caused by residual material stuck on the stamping die which can not be cleaned in time. During the next stamping, the residual material will pad a small pit on the parts and is randomly distributed on the surface of the parts, as shown in Figure 3c.
4. The edging is caused by excessive grinding of the edge of the parts due to the improper placement of the parts or the deviation of the grinding wheel during the grinding process, and it is mostly distributed on the edge of the parts, as shown in Figure 3d.
5. The scratch is caused by a sharp scratch or metal dust falling into the mold. When the parts is processed, a long and narrow curve is drawn on the surface and randomly distributed on the surface of the parts, as shown in Figure 3e.

Defects can be divided into center panel area defects and edge annular area defects according to the distribution position, and the distribution position of defects is irregular; moreover, due to the defects being small, it is difficult to detect and identify all defects under a commonly used light source.

## 5. Detection Steps and Algorithms

Because of the diversity and minuteness of the surface defects of stamping parts, all the defects cannot be detected and identified only by the common single direction lighting conditions, but when the light from different directions successively irradiates on the parts surface, all types of defects will be easier to detect and identify. In this paper, we will use the lighting method of combining a plane light source and multi-angle light source to collect the parts surface image. The process of image acquisition is as follows: when the plane light source is turned on, the plane light source image can be obtained. The image is mainly used to extract the region of interest about the parts and carry out defects detection and recognition for the first time; when the multi-angle light source is turned on alternately, the multi-angle light source images can be obtained and the images are used for further defects detection and recognition. In order to achieve the detection accuracy and improve the robustness of the system, the multi angle image acquisition of parts is carried out with four sets of lighting angles in this paper.

### 5.1. Segment the Region of Interest of Parts

**Figure 4:**
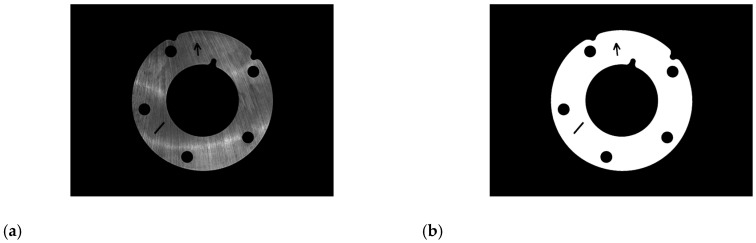
The region of interest for parts: (**a**) before segmentation; (**b**) after segmentation.

In addition to the region of interest of the parts, the original image also includes a black background, if defects detection and identification are carried out directly, the calculation time will be increased. Based on the plane light source image, the region of interest of the parts is extracted by template matching [25,26]:

1. Making template: before testing the same batch of parts, select a qualified part of the batch firstly, and take an image under the plane light source, as shown in Figure 4a. The parts region is segmented from the background by global threshold segmentation, and the region of interest is obtained, which is the batch parts template, as shown in Figure 4b.
2. Matching the parts region: according to the shape of the template, the template is used to search the parts region, and make the template overlap with the target region by translating and rotating the radiation transformation of the template. In order to improve the calculation speed, 9-layer pyramid structure is used to accelerate the matching parts region [27].
3. Segmenting region of interest: undertaking a bit and between the parts image and template after affine transformation, and then, setting the gray value of other regions except the region of interest of parts to zero. Extracting non-zero region is the region of interest of parts. Testing the same batch of parts only once to make a template, after which test repeat (2) (3) steps.

### 5.2. Detection and Extraction of Surface Defects of Parts

#### 5.2.1. Plan Light Source Image Defects Detection

Firstly, image preprocessing is carried out for the region of interest of the parts: the image is converted into gray image, and the gray image is opening operation (first erode and then dilate) to remove small areas, disconnect narrow connections, eliminate noise, smooth the boundary of the region without changing the area.

**Figure 5:**
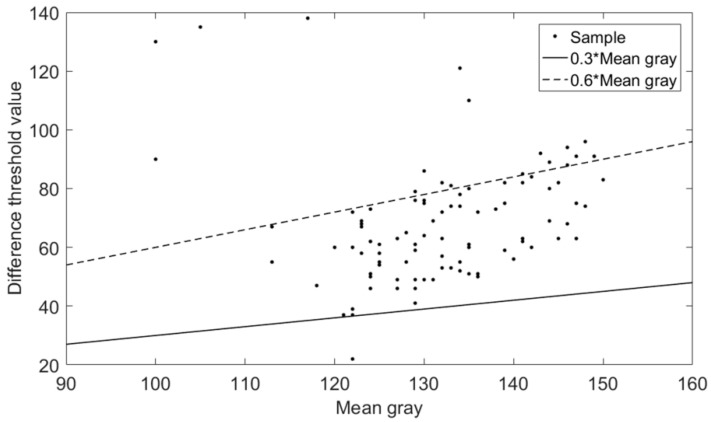
The selection of difference coefficient.

Secondly, the grayscale abnormal region is extracted from the preprocessed image. Due to gray abrupt change of defective region on the surface of the parts, a differential threshold segmentation method is used to extract grayscale abnormal region. The specific operation is as follows: calculate the average gray value of the parts region. If the difference between the gray level of a pixel in the original image and the average value is greater than the set threshold value, the pixel is the grayscale abnormal pixel point, and the set of grayscale abnormal pixel points is the grayscale abnormal region. The setting of the two thresholds is related to the gray average value of the parts region and also affected by external factors such as light source intensity. A simple mathematical model for selecting the thresholds:

(1) $$T_{h}=K\times M_{\mathrm{GS}}$$

where *T*_h_ is threshold value, *K* is difference coefficient, *M*_GS_ is the mean gray. According to the experiment on 100 defective parts as shown in Figure 5, where y-coordinate is the difference between gray value of defective region and average gray value of parts region. The region with difference greater than *T*_h_ is identified as grayscale abnormal area.

**Figure 6:**
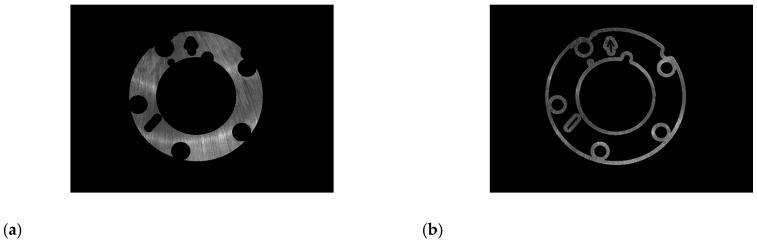
Division of parts: (**a**) the central panel region; (**b**) the central panel region.

Finally, the grayscale abnormal region extracted contains noise, so the grayscale abnormal regions are filtered by feature parameters. Area feature is one of the most commonly used features to extract a region, and the area of noise is small, so it can be quickly and accurately eliminated by using area feature. According to the location distribution law of defects, the parts region is divided into center panel region and edge annular region before area filtered. The method is as follows:

1. After eroding the region of parts, which the central panel region shown in Figure 6a;
2. The edge annular region can be obtained by making a difference between the parts region and the center panel region, as shown in Figure 6b.

After region division, the grayscale abnormal region should be located. Regional positioning can be achieved by intersecting gray anomaly regions with center panel region and edge annular region respectively. The gray level anomaly region which only intersect with the edge annular region are classified as Category *A* regions, while those which intersect only with the center panel region or with the center panel region and the edge annular region are Category *B* regions. Set two area thresholds *S*_1_ and *S*_2_ (*S*_1_ and *S*_2_ will be affected by the distance between the camera and the parts) and extract the regions with area greater than *S*_1_ in Category *A* region and those with area greater than *S*_2_ in Category *B* region as defective regions. Finally, the defect region is extracted by closing operation (first dilate and then erode), which can fill the voids and smooth edges to make the defect extraction better.

#### 5.2.2. Multi-Angle Light Source Image Defects Detection

**Figure 7:**
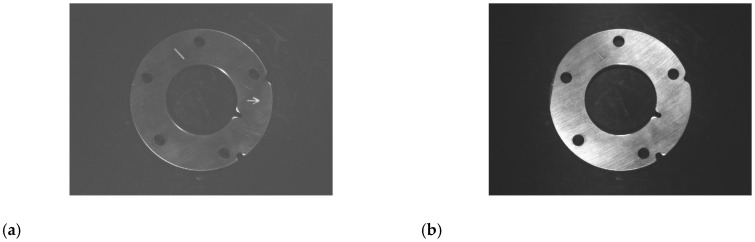
The image of different grinding marks angle: (**a**) the grinding marks angle closes to 90 degree; (**b**) the grinding marks angle closes to 0 degree.

After preliminary detection and recognition of planar light source image, it is found that there are several kinds of defect that can not be completely extracted or detected, so further defects detection and recognition are rEquation uired by multi-angle light source image. During grinding of parts, grinding marks will be generated on the surface of parts. Under multi-angle illumination, the reflecting effect of grinding marks is obviously affected by the angle of grinding marks and light source (hereinafter referred to as grinding marks angle). When the ray of light is close to parallel with the direction of grinding marks, the parts region in the image is too dark as shown in Figure 7a; when the ray of light is close to perpendicular to the direction of grinding marks, the parts region is too bright as shown in Figure 7b; others the brighter the parts region is as the angle approaches 90 degrees. Therefore, when choosing a multi-angle light source lighting scheme, the reflection characteristics of the region of interest should be taken into account in order to achieve the best lighting effect.

**Figure 8:**
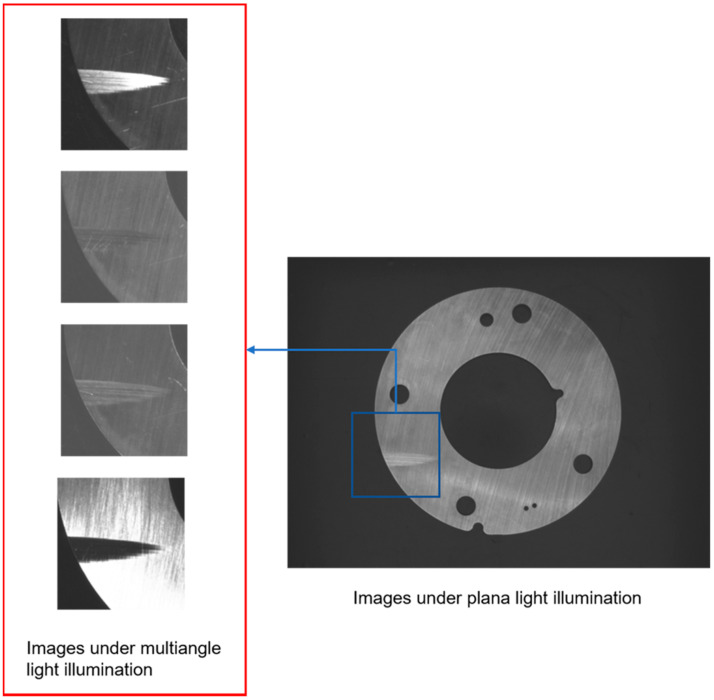
The parts under multiangle illumination.

**Figure 9:**
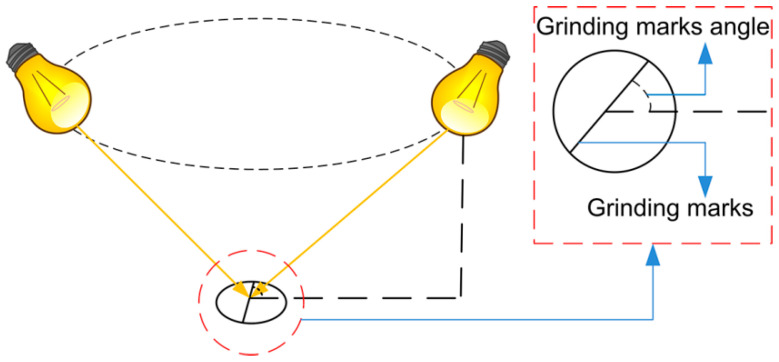
The angle of grinding marks.

When the image is taken by multi-angle light source, it is difficult to identify and segment defective regions only by detecting grayscale abnormal regions because the image is greatly affected by grinding marks. As shown in Figure 8, there is a cliff-like brightness change between the defective region and surrounding region in the multi-angle light source image, so there will be a clear and smooth edge line. This edge line will be an important reference for defect detection and a defect detection method based on edge line is proposed. The specific operation is as follows:

The first step calculates the grinding marks angle, which is the angle of the grinding marks and the ray of light. It is specified as the clockwise angle between the projection of light ray on the surface of the parts and the grinding marks, as shown in Figure 9. The calculation process is as follows: firstly, gradient edge extraction is performed on the plane light source image, and secondly, grinding marks region filtering is carried out by unEquation ual axis feature *A*_n_:

(2) $$A_{n}=R_{a}/R_{b}$$

**Figure 10:**
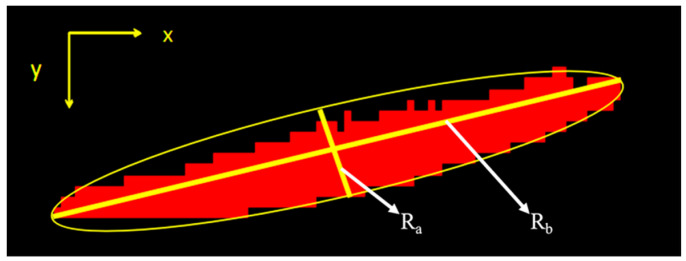
External ellipse.

**Figure 11:**
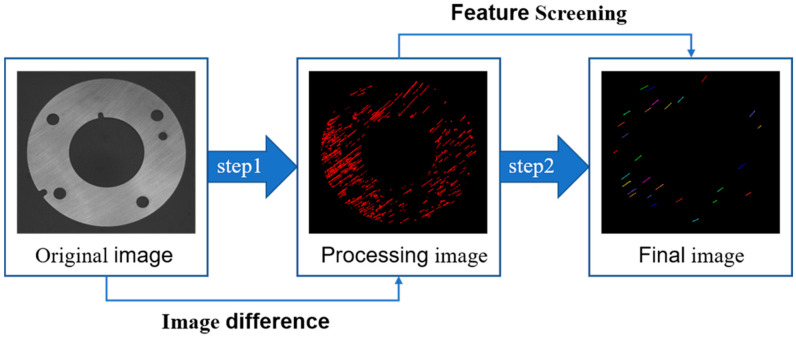
The extraction process of grinding marks.

*R*_a_ is the long axis of the Equation uivalent ellipse, *R*_b_ is the short axis of the Equation uivalent ellipse, the Equation uivalent ellipse of the connected domain is shown in Figure 10. The process of extracting the grinding marks is shown in Figure 11. Since the installation position and angle of the multi-angle light source are unchanged, the running direction of the conveyor belt is set as the datum line. Finally, the average angle alpha between the long axis of the Equation uivalent ellipse and the datum line in all the grinding marks regions after screening is calculated, which covers the range of [0°, 180°], and the angle calculation formulas for each angle light source are shown in Table 2, where *β*_a_, *β*_b_, *β*_c_ and *β*_d_ are the grinding marks angles illuminated by multi-angle light sources in groups *a*, *b*, *c* and *d* (grouping is shown in Figure 2a).

The second step determines the optimal exposure time, which is the camera exposure time when the average gray level (hereinafter referred to as average gray level) of the region in the qualified parts image is around 110. When the average gray level of the parts region is around 110, it means that the parts region has no overall phenomenon of too high or too low gray level, which can better reflect the image details.

**Figure 12:**
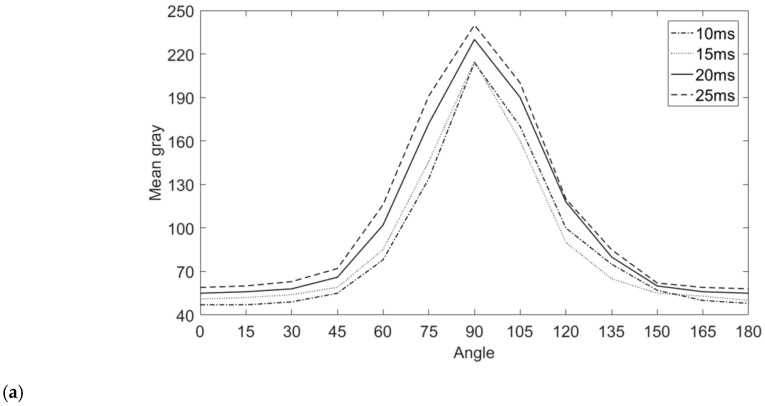

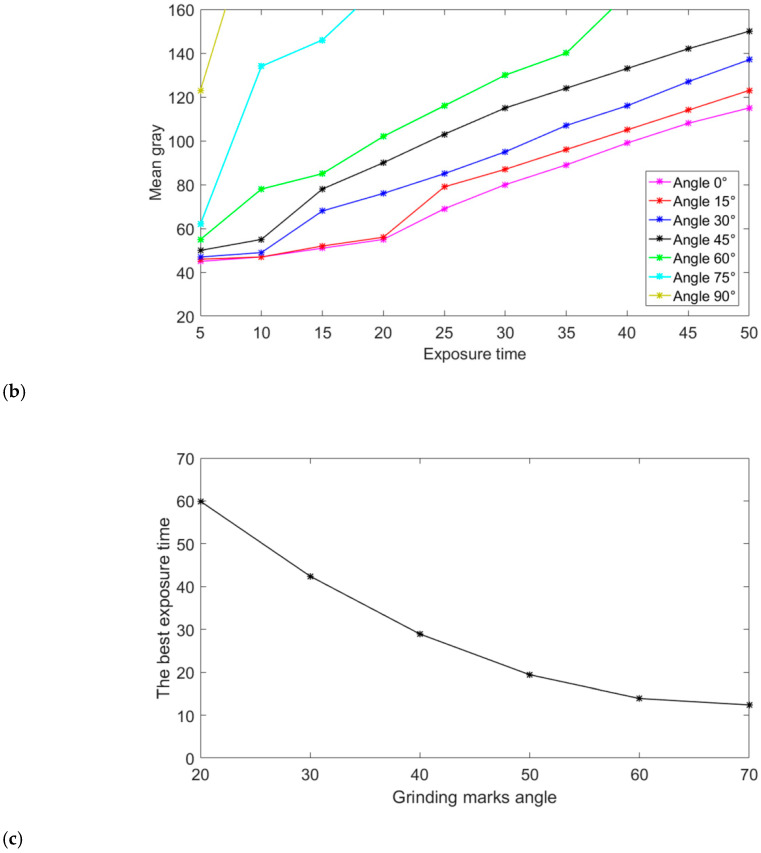
The data for selecting the best appropriate exposure time: (**a**) the relationship of angle and mean grayscale; (**b**) the relationship of exposure and mean grayscale; (**c**) the relationship of grinding marks angle and the best exposure time.

First of all, understand the reflection law of different grinding marks angles, and then determine the best exposure time in each angle range according to the average gray level of the parts region. Above all, keep the exposure time unchanged, open a group of angle light sources, take the qualified parts, rotate the grinding marks angle 180 degrees from 0 degrees, take a picture every 15 degrees and record the average gray level, then change the exposure time to do the above operations, and finally connect each point under each exposure time with a smooth curve to obtain the grinding marks angle reflectance curve, as shown in Figure 12a. By analyzing the curves and combining the experiments, the following conclusions can be drawn: because each group of angle light sources is symmetrical, the reflectance curve is axially symmetrical at 90 degrees; when the grinding marks angle is between 0 degrees and 20 degrees, most of the light is reflected outside the lens by the grinding marks, resulting in low average gray level as shown in Figure 7a. When the grinding marks angle is between 70 degrees and 90 degrees, most of the light is reflected into the lens by the grinding marks, which leads to the high average gray level as shown in Figure 7b, the light spots appear in the image. When the grinding marks angle is between 20 degrees and 70 degrees, the average gray level approaches the quadratic function increasing rule as the grinding marks angle increases.

According to the reflection law of the grinding marks angle, the best exposure time of each group of grinding marks angle is determined. The following experiments are carried out to keep the grinding marks angle unchanged, change the exposure time and calculate the average gray value of the parts region. According to the experimental data, we can obtain the following conclusions: when the grinding marks angle is between 0 and 20 degrees, the average gray level is low and insensitive to the angle change, so we can directly choose a large exposure time, such as 50ms; when the grinding marks angle is between 70 and 90 degrees, the image appears bright spots, and the average gray level changes irregularly and the average gray level is high within this range, so we can directly choose the low exposure time, Such as about 5ms. When the grinding marks angle is between 20 and 70 degrees, keep the grinding marks angle unchanged, establish the relationship between the exposure time and the average gray level as shown in Figure 12b, and select the exposure time when the average gray level is closest to 110. The result is shown in Figure 12c.

The third step is edge extraction. The algorithm of edge detection is mainly based on the first-order and second-order derivatives of image strength, but the derivatives are usually very sensitive to noise, so it is necessary to use filters to improve the performance of edge detection related to noise. In this paper, a Gauss filter is used. The size of the Gauss convolution kernel will affect the effect of edge extraction. The larger the size of convolution kernel is, the better the effect of noise removal is, but the edge will also be blurred, so the appropriate Gauss convolution kernel should be selected (the Gaussian convolution kernel in this paper is (5,5)). Calculate the image gradient intensity and gradient direction (the gray gradient is the gray value difference between two diagonally adjacent pixels in this paper), as shown in Equation (3):

(3) $$\begin{array}{l} G_{x}\left(x,y\right)=f\left(x,y\right)-f\left(x-1,y-1\right)\\ G_{y}\left(x,y\right)=f\left(x-1,y\right)-f\left(x,y-1\right)\\ G=\sqrt{G_{x}^{2}\left(x,y\right)+G_{y}^{2}\left(x,y\right)}\\ \theta =\arctan(G_{y}\left(x,y\right)/G_{x}\left(x,y\right)) \end{array}$$

**Figure 13:**
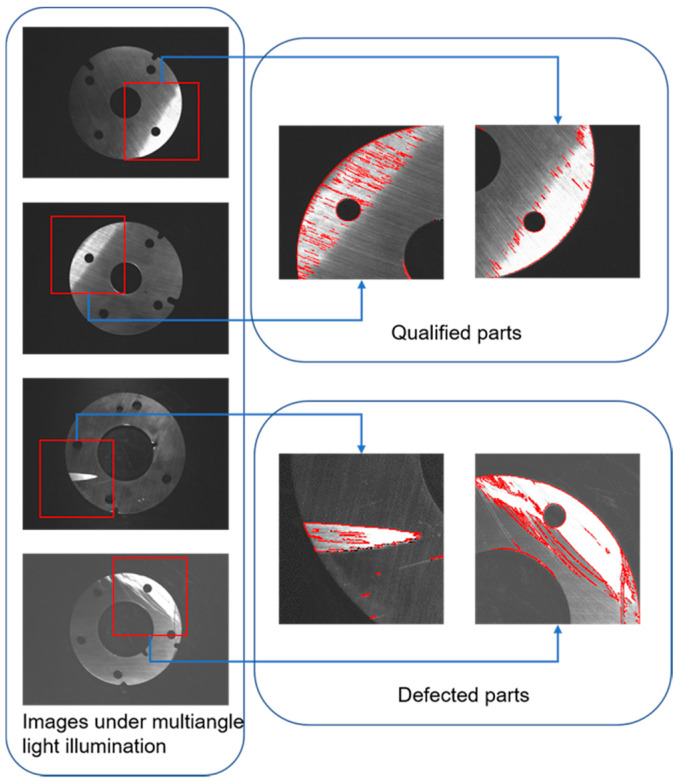
Edge extraction of gray anomaly region.

In Equation (3), *f*(*x*, *y*) is the gray level of pixel (*x*, *y*), *G*_x_(*x*, *y*) and *G*_y_(*x*, *y*) are the gradients of pixel (*x*, *y*) along the *X* and *Y* coordinate axes, *G*(*x*, *y*) is the gradient intensity of pixel (*x*, *y*), and *θ* is the gradient direction angle of pixel (*x*, *y*). Set the high and low threshold values (in this paper, the upper threshold is 40 and the lower threshold is 20). When the gradient intensity of a pixel (*x*, *y*) exceeds the high threshold value, the pixel remains as an edge point; if the gradient intensity of a pixel (*x*, *y*) is less than the low threshold value, it is excluded; if the gradient intensity of a pixel (*x*, *y*) is between the upper threshold and the lower threshold, it will be saved when it connects with a pixel of the higher upper threshold. There may be interference in the extracted edge regions. As shown in Figure 13 for defected parts, the real rEquation uired edge can be extracted by structural factor. The definition of structural factor is shown in Equations (4)–(6):

(4) $$A_{n}=R_{a}/R_{b}$$

(5) $$B_{u}=\pi R_{a}R_{b}/A$$

(6) $$B_{u}=\pi R_{a}R_{b}/A$$

In Equations (4)–(6), *R*_a_ and *R*_b_ are the long axis and the short axis of the Equation uivalent ellipse, *A* is the area of the connected domain, and *A*_n_ is the unEquation ual axis, which reflects the shape of the connected domain by the ratio of the Equation uivalent ellipse long axis and the short axis in the connected domain. *B*_u_ is the Bulkiness, which reflects the dispersion of the connected domain by the ratio of the area of the Equation uivalent ellipse and the area of the connected domain. Bulkiness is either “loose” or “compact”. *S*_f_ is the structure factor. Defect edges are characterized by “narrow” and “compact”, so their structural factor values are usually large, where the desired edges can be screened by setting a large structural factor threshold. The Equation uivalent ellipse of the connected domain is shown in Figure 10.

The fourth step is defects identification, to determine whether the grayscale abnormal region is defective by edge line around the grayscale abnormal region. The detection method is as follows: expanding the edge to form an edge region; if there is an intersection between the two region, it means that there is an edge line around the grayscale abnormal region, which conforms to the characteristics of the defect region; conversely, exclude this region.

### 5.3. Misjudgment Check

As shown in Figure 13, the image of qualified parts is collected under a multi-angle light source, there are no defects on the surface of the parts, but a grayscale abnormal region appear on the image due to grinding marks reflections. In order to prevent such regions from being misjudged as defects, a misjudgment check should be carried out on the detected “defect region” at the end of the defects detection. This paper carries out a misjudgment check according to the direction of the edge line. The bright region formed by grinding marks reflections is due to the fact that the light runs perpendicular to the grinding marks and is reflected into the lens. Because of the uneven depth of the grinding marks, the gray level between this type of super-bright region and surrounding region decreases rather than changes abruptly, and usually does not form edges. For individual edge-reflecting regions, the edges are usually discontinuously serrated, and the direction is nearly vertical to the direction of gray anomaly region, as shown in Figure 13 for qualified parts. Edge grinding defects are usually caused by strong relative movements between the parts and the die or other parts, so that the region is oriented in the same direction as its edge. The direction of the region refers to the long axis direction of the external ellipse in the region, as shown in Figure 10. Therefore, the clockwise angle between the direction of the gray anomaly region and the direction of the edge of the gray anomaly region can be used to determine whether the area is a real defect. If the angle is large (the angle is 45 degree in this paper), the area is not a defects region.

**Figure 14:**
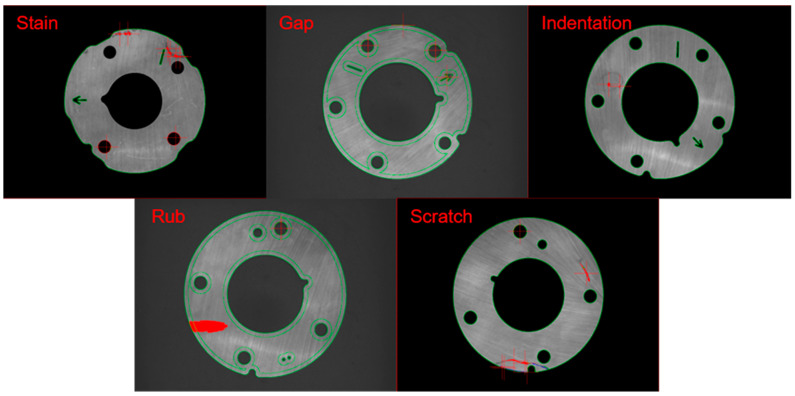
Defects detection for the parts.

Through the above process, we can obtain the detection and extraction effect of five types of defects as shown in Figure 14.

## 6. Experimental Verification and Results

**Figure 15:**
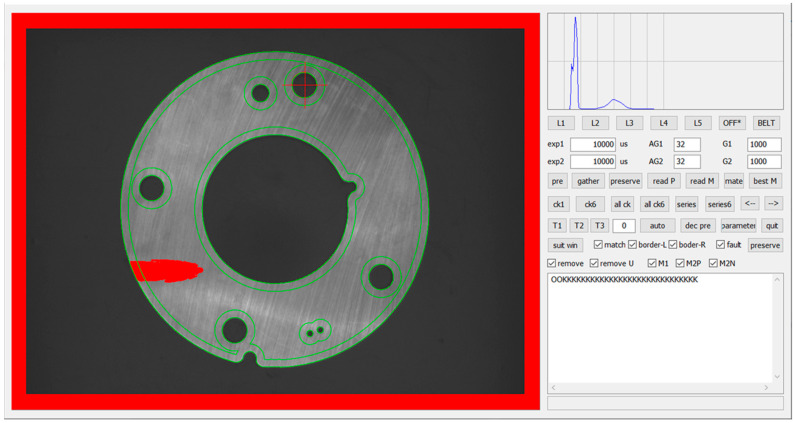
Software system interface.

In order to verify the detection method proposed in this paper, an experimental platform is built as shown in Figure 1. Microsoft Visual Studio 2013 is used to write the detection software, and the interface is shown in Figure 15. Experimental parameter setting: in the plane light source detection, the dynamic threshold segmentation offset is 30, and the erosion radius of the segmented part region is 20 pixels; in the multi angle light source detection, the edge dilation radius is 9 pixels, and the *K* value is 0.5 and 1.5 respectively.

The tested object valve plate is a kind of flat metal part, with various valve plate shapes and diameters ranging from 10 cm to 30 cm. We set up two groups of experiments: the defect group, in which 50 parts with defects are selected, 10 samples for each defect (each sample req uires takes five images, so the system needed to process 250 images in this group). This group mainly tests the system to extract various defects, preventing nonconforming products from being not detected due to a type of detect not be extracted. The experimental results are shown in Table 3. In the random group, 150 parts are selected randomly, in which qualified products and unqualified products are mixed (every sample has two sides and each sides req uires takes five images, so the system needed to process 1500 images in this group). This group imitates the actual testing environment and check the system can detect the nonconforming parts. The results of the experiment are shown in Table 4.

It is found that three defects regions (misrun, indentation, edging) can be completely extracted without missing inspection. However, compared with manual detection, the products with stains and scratches can be detected as unqualified products, but the defect area can not be extracted completely. The reasons for this phenomenon are: the size of stain is uncertain, the transparency of several stains is high, the contrast is low, and the grayscale difference is within 20, the scratch depth is uneven, and the tip area cannot be completely extracted.

In general, the accuracy of this method can reach 100% for the defects detection of grinding flat parts, in other words, the system can choose the non-conforming parts. But in some cases, it can not completely extract the stains and scratches’ contrast with manual inspection, and the extraction accuracy can reach 98.6%. It is worth noting that, as a substitute for manual screening of the unqualified products of grinding flat parts, the detection system has reached the rEquation uirement of no missing inspection and can be applied to the actual factory detection process.

## 7. Conclusions

This paper introduces five kinds of defects in the process of stamping and grinding flat parts, and introduces in detail methods of defect detection under two kinds of lighting methods and elimination of misjudgment. From software design to platform construction, a machine vision-based surface defect detection system for stamping and grinding flat parts is developed, and the performance of the system is verified by experiments. The experiment shows that the hardware structure of the system is reliable and the work is stable, which meets the rEquation uirements of defect detection in the production process of parts. The system can choose the non-conforming parts, and the accuracy of the system can reach 100%. However, in some cases, compared with manual detection, the products with stains and scratches can be detected as unqualified products, but the defect area cannot be extracted completely, and the extraction accuracy can reach 98.6%. In the process of practical production and application, it can greatly improve production efficiency and save labor cost, which has great theoretical and practical value.

## Figures and Tables

**Table 1:** Comparison to other works.

	Method	Application	Result
Min [15]	light-emitting diode (LED) auxiliary light source the contour of the direction chain code	Rail surface defects	Speed of 2 m/s
Chen [16]	a dual-lighting system variance transform binary large object analysis	Steel ball	Detection rate of 99.94%
Zhang [19]	two image processing algorithms domain filtering methods	Magnetic ring	Recognition rate of 92.5% and 91.5%
This paper	combine plane illumination and multi-angle illumination modes the image-processing algorithms	Stamping and grinding flat parts	Detection rate of 98.6%

**Table 2:** Formula for calculating grinding marks angle.

Angle (°)	Calculation Formula
*β*_a_	$\begin{array}{cc} 90{}^{\circ}-\alpha & 0{}^{\circ}<\alpha \leq 90{}^{\circ}\\ \alpha -90{}^{\circ} & 90{}^{\circ}<\alpha \leq 180{}^{\circ} \end{array}$
*β*_b_	$\begin{array}{cc} 45{}^{\circ}-\alpha & 0{}^{\circ}<\alpha \leq 45{}^{\circ}\\ \alpha -45{}^{\circ} & 90{}^{\circ}<\alpha \leq 180{}^{\circ}\\ 225{}^{\circ}-\alpha & 135{}^{\circ}<\alpha \leq 180{}^{\circ} \end{array}$
*β*_c_	$\begin{array}{cc} \alpha & 0{}^{\circ}<\alpha \leq 90{}^{\circ}\\ 180{}^{\circ}-\alpha & 90{}^{\circ}<\alpha \leq 180{}^{\circ} \end{array}$
*β*_d_	$\begin{array}{cc} \alpha +45{}^{\circ} & 0{}^{\circ}<\alpha \leq 45{}^{\circ}\\ 135{}^{\circ}-\alpha & 45{}^{\circ}<\alpha \leq 135{}^{\circ}\\ \alpha -135{}^{\circ} & 135{}^{\circ}<\alpha \leq 180{}^{\circ} \end{array}$

**Table 3:** The results for defect group.

Defects Category	Stain	Misrun	Indentation	Edging	Scratch
Number	10	10	10	10	10
Number of successful detections	10	10	10	10	10
Number of complete extractions	91	10	10	10	8
Rate of successful detections (%)	100	100	100	100	100
Rate of complete extractions (%)	90	100	100	100	80

**Table 4:** The results for random group.

Number	Number of Successful Detections	Number of Complete Extractions	Rate of Successful Detections	Rate of Complete Extractions
150	150	148	100%	98.6%
